# ZnO@ZIF-8 Nanoparticles as Nanocarrier of Ciprofloxacin for Antimicrobial Activity

**DOI:** 10.3390/pharmaceutics15010259

**Published:** 2023-01-11

**Authors:** Bruno Altran Costa, Marina Paiva Abuçafy, Thúlio Wliandon Lemos Barbosa, Bruna Lallo da Silva, Rafael Bianchini Fulindi, Guilherme Isquibola, Paulo Inácio da Costa, Leila Aparecida Chiavacci

**Affiliations:** 1 School of Pharmaceutical Sciences, São Paulo State University (UNESP), Araraquara 14800-903, Brazil; 2 Institute of Chemistry of Araraquara, São Paulo State University (UNESP), Araraquara 14800-900, Brazil; 3 Departments of Clinical Analysis, São Paulo State University (UNESP), Araraquara 14800-903, Brazil

**Keywords:** metal-organic framework, ZnO, nanoparticles, antimicrobial activity

## Abstract

Numerous antimicrobial drugs have been prescribed to kill or inhibit the growth of microbes such as bacteria, fungi, and viruses. Despite the known therapeutic efficacy of these drugs, inefficient delivery could result in an inadequate therapeutic index and several side effects. In order to overcome this adversity, the present study investigated antibiotic drug loading in zeolitic imidazolate frameworks (ZIFs), in association with ZnO nanoparticles with known antimicrobial properties. In an economic synthesis method, the ZnO surface was first converted to ZIF-8 with 2-methylimidazole as a ligand, resulting in a ZnO@ZIF-8 structure. This system enables the high drug-loading efficiency (46%) of an antimicrobial drug, ciprofloxacin, within the pores of the ZIF-8. This association provides a control of the release of the active moieties, in simulated body-fluid conditions, with a maximum of 67% released in 96 h. The antibacterial activities of ZnO@ZIF-8 and CIP-ZnO@ZIF-8 were tested against the Gram-positive *Staphylococcus aureus* strain and the Gram-negative *Pseudomonas aeruginosa* strain, showing good growth inhibition. This result was obtained by combining ZnO@ZIF-8 with ciprofloxacin in a minimal inhibitory concentration (MIC) that was 10 times lower than ZnO@ZIF-8 for *S. aureus* and 200 times lower for *P. aeruginosa*, suggesting that CIP-ZnO@ZIF-8 may have potential application in prolonged antimicrobial treatment.

## 1. Introduction

Infectious diseases caused by invasive pathogens continue to pose serious mortality and morbidity threats to global health, even as medical care has improved around the world. In addition, the crisis is impacted by multidrug-resistant bacterial strains caused by the abuse and misuse of antibiotics. Antibiotic resistance is estimated to cause 700,000 deaths annually worldwide, and that number is expected to rise to over 10 million deaths per year by 2050 [1]. 

Antibacterial nanotherapeutic strategies have been highlighted in the literature ac-cording to their mechanisms. Recently, Wang et al. reviewed nanocarriers for antibacterial agents, including inorganic nanoparticles (NPs), polymeric NPs, and metal-organic framework structures (MOFs) that exhibit intrinsic antibacterial activity and may lead to more potent activity, as well as for decreasing the occurrence of antibiotic resistance [2]. 

Among the inorganic antimicrobial agents, zinc oxide (ZnO) has excellent advantages due to its antimicrobial property, showing activity against Gram-positive and Gram-negative bacteria, in addition to having greater durability and lower toxicity [3]. 

The antibacterial action of ZnO NPs can be described as having three main mechanisms: (1) production of reactive oxygen species (ROS), (2) release of Zn^2+^ ions, and (3) interaction of NPs with the cell wall. These mechanisms can cause damage to cellular components, facilitating the cellular internalization of ZnO NPs. Another determining factor for the antimicrobial action of ZnO NPs is their size. The smaller the size of the NPs is, the greater the surface area of these NPs is, and, consequently, the greater the interaction with the cellular components of the bacteria is, so the antimicrobial activity becomes greater and faster [4]. On the other hand, ZnO NPs have exceptional physicochemical characteristics, such as their luminescent properties, making it possible to use these NPs to monitor the biodistribution of drugs in the body in addition to the presence of hydroxyl terminals on the surface of the NPs, allowing for their conjugation with different molecules [5,6].

Porous materials, such as MOFs, have great potential as drug nanocarriers. MOFs are a class of coordination polymers composed of metallic and organic ligands, capable of forming a highly porous crystalline structure with an adjustable shape and size [7,8]. Imidazole zeolitic structures (ZIF-8) are a subgroup of MOFs comprising tetrahedrally coordinated Zn^2+^ linked by a 2-methyl imidazole (2-MeIM) organic linker. Its favorable physicochemical properties, such as pore size, allow it to carry high drug loading and an efficient controlled release [9,10]. Based on these characteristics, ZIF-8 was selected as the drug-carrier material for the present study.

The synergistic effect of combining ZnO NPs with antibiotics, including ciprofloxacin (CIP), had the greatest synergistic effect against *Staphylococcus aureus* and *Pseudomonas aeruginosa* [11,12,13]. Thus, using ZnO@ZIF-8 is an advantageous approach because it can combine the properties of each material to build a structure with different applications. This study demonstrated a new synthesis for the formation of ZnO@ZIF-8 MOF, showing the advantages of using this system as a nanocarrier of drugs. Furthermore, it is possible to observe how this system can be used for antibacterial application by incorporating ZnO@ZIF-8 with CIP. This approach is innovative and allows for the construction of a material with optimized antibacterial properties and a controlled release. 

## 2. Materials and Methods

### 2.1. Materials

Ciprofloxacin raw material, Zinc acetate dihydrate, Lithium hydroxide, (3-Glycidyloxypropyl) trimethoxysilane (GPTMS), and 2-Methylimidazole (2-MeIM) were purchased from Sigma-aldrich™ (Cotia, Brazil). Hydrochloric acid was obtained from Quimex™ (San Martín de Porres, Peru). Water was purified with a Milli-Q Plus™ system (Millipore Corporation, Bedford, MA, USA). Absolute ethanol was supplied by Synth™ (Diadema, Brazil). Monobasic sodium phosphate and sodium hydroxide for PBS bupher pH 7.4 were provided by Quimis™ (Diadema, Brazil).

### 2.2. Methods

#### 2.2.1. Synthesis of Colloidal Suspensions of ZnO NPs

The ZnO nanoparticles were obtained from a synthesis proposed by Spanhel and Anderson [14], with some modifications. The precursor was prepared through an ethanolic solution of zinc acetate (Zn(CH_3_COO)_2_·2H_2_O) (0.1 mol L^−1^) at 80 °C and refluxed for 2 h under N_2_ atmosphere. The solution was diluted (0.05 mol L^−1^) and stored in the freezer.

The colloidal suspension of ZnO NPs was made with lithium hydroxide (LiOH) as a hydrolysis agent, in which the amount was calculated to reach a hydrolysis ratio (r = [OH^+^]/[Zn^2+^]) equal to 1.0. The reaction was carried out in an ultrasonic bath for one hour at 50 °C. At the end of this reaction, a colloidal suspension of ZnO NPs was obtained.

Surface modification of the ZnO NPs was performed by binding (3-Glycidyloxypropyl) trimethoxysilane (GPTMS) to the surface of the ZnO nanoparticles to make them stable in water and, thus, allow biological applications. For this, 0.1 mol L^−1^ of GPTMS and 0.2 mol L^−1^ of LiOH were added to 10 mL of ZnO suspension, thus promoting a new step of hydrolysis and condensation. The reaction took place in an ultrasound bath at room temperature for 30 min at 35 °C. At the end of the reaction, it was possible to observe the formation of a white precipitate, which was separated by centrifugation for 10 min at 4000 rpm and dried at room temperature. The collected powder was dispersed in water.

#### 2.2.2. Synthesis of ZIF-8 NPs

ZIF-8 particles were synthesized, as previously described [15], with appropriate adjustments. First, a solution of Zn(NO_3_)_2_·6H_2_O (0.29 g) in 20 mL of methanol was rapidly poured into a solution of de 2-methylimidazole (2-MeIM) (0.69 g) in 20 mL of methanol under stirring with a magnetic bar for 1 h. The nanocrystals were collected by centrifugation and washed with fresh ethanol 3 times. The sample was dried at 50 °C in a vacuum.

#### 2.2.3. Synthesis of ZnO@ZIF-8 Nanoparticles

The synthesis of ZnO@ZIF-8 NPs was based on the methodology described by Wang et al. [16], with some modifications. First, 20 mL of 2-MeIM solution (0.24 mol L^−1^) was added dropwise to the aqueous suspension of ZnO that had been previously synthesized (Section 2.2.1) under constant stirring at room temperature for 1 h. After reaching the required time, the resulting material was centrifuged for 15 min at 6000 rpm and then washed twice in ethanol. Finally, the material was dried and activated in a vacuum oven for 24 h at 75 °C.

#### 2.2.4. Characterization

X-ray diffraction (XRD) was carried out to evaluate the crystal states of samples using an X-diffractometer q™ (Rigaku, Neu-Isenburg, Germany). All samples were measured at 60 mA and 40 kV, with scan range of 2–75° and scan interval of 0.02°. Scanning electron microscopy (SEM) was carried out to evaluate the morphology by differences in the appearances of drug powders and nanocrystals, crystalline habits, cluster-formation tendencies, and visual aspects of nanocrystals. The samples were carbon-coated to about 15 nm thickness using a Sample Sputter Coater SCD 050™ (Bal-Tec, Wallruf, Germany), for 70 s. After that, SEM pictures were taken on an scanning electron microscopy SM300™ (TOPCON, Singapore, Singapore) instrument. FTIR analysis was carried out using an infrared spectrophotometer with Fourier-transform Shimadzu™ Affinity (Kyoto, Japan), with spectra realized at room temperature in the range of 4000–370 cm^−1^. Thermal analysis was carried out by thermogravimetric analysis (TGA), using thermogravimetric analyzer TGA 50 Shimadzu™ (Kyoto, Japan) at a heating rate of 20 °C min^−1^ and at an interval range from 25 to 800 °C in nitrogen flow of 100 mL min^−1^. It used samples of 8 mg. Zeta potential (ξ) value was analyzed on a Zetasier Nano ZS™ photon correlation spectroscopy (Malvern Instruments, Malvern, UK). Measurements were performed in triplicate from fresh nanocrystal suspensions at room temperature (25 °C) as well as dry and redispersed nanocrystal suspensions. N_2_ sorption isotherms were obtained at 77 K using an ASAP porosimeter (accelerated surface area and porosimetry system, model 2013; Micromeritic, Norcross, GA, USA), connected to a computer. The samples were evacuated at 120 °C under primary vacuum. Brunauer-Emmett-Teller (BET) surface area and pore volume were estimated at a relative pressure ranging from 0.05 and 0.25.

#### 2.2.5. Ciprofloxacin Loading and Release

The drug was loaded by soaking 15 mg of ZnO@ZIF-8 in 30 mL of an ethanolic solution of CIP (0.5 mg mL^−1^) under stirring at room temperature in a shaker, protected from light. Aliquots were collected at different times (12 h, 24 h, and 48 h) to evaluate the best entrapment efficiency. The drug in the supernatant was measured by UV–vis spectroscopy at 273 nm, and the drug entrapment efficiency (*EE%*) was calculated on the basis of Equation (1):

(1) $$\mathrm{Entrapment}\;\mathrm{efficiency}\;\left(\mathrm{EE}\%\right)=\frac{\mathrm{weight}\;\mathrm{of}\;\mathrm{CIP}\;\mathrm{in}\;\mathrm{ZnO}@\mathrm{ZIF}-8}{\mathrm{weight}\;\mathrm{of}\;\mathrm{CIP}\;\mathrm{fed}\;\mathrm{initially}\;}\times 100$$

The CIP release profile was acquired by using simulated physiological medium (PBS buffered solution) at pH 7.4, representing the physiological pH. CIP-ZnO@ZIF-8 was suspended in PBS solution (30 mL) and incubated at 37 °C under constant stirring in triplicate. Aliquots were removed after 0.5, 1, 2, 3, 4, 6, 8, 12, 24, 36, 48, 72, and 96 h and quantified, indirectly, by UV–vis spectroscopy at 273 nm. 

#### 2.2.6. Antibacterial Potential of ZIF-8, ZnO, ZnO@ZIF-8 and CIP-ZnO@ZIF-8

Minimum inhibitory concentration (MIC) was performed to assess the influence of NPs on bacterial metabolism using an XTT assay (2,3-Bis(2-methoxy-4-nitro-5-sulfophenyl)-5-[(phenylamino)carbonyl]-2H—tetrazolium), according to the study carried out by Peeters et al. [17]. In summary, the bacterial strains of *S. aureus* (ATCC 25923) and *P. aeruginosa* (ATCC 27853) were standardized according to the Clinical and Laboratory Standards Institute (CLSI) protocol M7-A10. A microdilution of base 2 in Müeller–Hinton broth of the studied samples, ZIF-8, ZnO, ZnO@ZIF-8, and CIP-ZnO@ZIF-8, was performed at initial concentrations of 1 mg mL^−1^ and 5 mg mL^−1^ to determine the MICs for *S. aureus* and *P. aeruginosa*, respectively. The bacterial concentration used was 10^4^ CFU mL^−1^. After an incubation period of 24 h at 37 °C, XTT in PBS (pH 7.4) was added to a final concentration of 5 mg mL^−1^, and menadione solution (0.4 mmol L^−1^) was prepared and added before starting the test. The reader, for the viability assay, was performed by adding the XTT–menadione solution to the microplate wells and, after a 5 h incubation period, the absorbance was read at 490 nm on BIO-RAD microplate PR4100 (Brussels, Belgium). Experiments were evaluated in triplicate, and data are expressed as mean ± SD.

## 3. Results and Discussion

### 3.1. Synthesis of ZnO@ZIF-8 NPs

**Figure 1:**
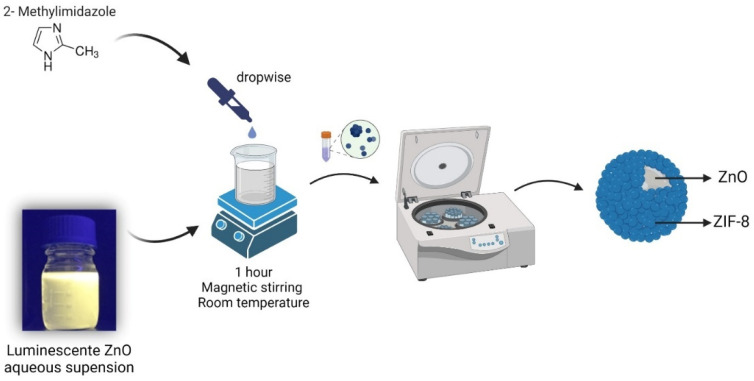
Schematic of ZnO@ZIF-8 NPs formation.

Figure 1 shows a schematic illustration of the developed material synthesis. After collecting the powder sample, luminescence remained when excited in a 365 nm lamp. The luminescence of ZnO is well-known in the literature. ZnO NPs are luminescent due to their quantum confinement [18]. As demonstrated in Figure 1, after ZIF-8 formation, the ZnO NPs remain luminescent. This result shows that the size of the ZnO NPs does not increase or barely increases in size, which is a very relevant result, since the size of the nanoparticles has already been shown to influence the antibacterial activity, and the smaller the nanoparticles are, the greater the activity is [19,20,21].

**Figure 2:**
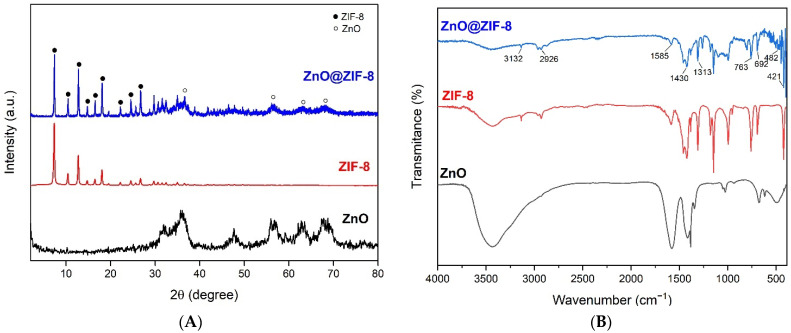
XRD patterns of ZnO NPs, ZIF-8 NPs, and ZnO@ZIF-8 NPs (**A**); FTIR spectra of ZIF-8 and ZnO@ZIF-8 NPs (**B**).

To confirm the formation of the ZnO@ZIF-8 conjugate structure, XRD and IR tests were performed. The XRD patterns (Figure 2A) of the materials showed the typical Bragg peaks of the ZIF-8 and ZnO topology, confirming that the desired crystal structures of both materials were obtained. The peaks were indicated by the filled circles in positions 7.31°, 10.32°, 12.86°, 14.66°, 16.60°, 18.03°, 22.17°, 24.45°, and 26.80° 2θ, corresponding to symmetry planes (011), (002), (112), (022), (013), (222), (114), (233), and (131), respectively. This, then, demonstrated the successful formation of ZIF-8 [22]. The diffraction peaks (empty circles), referring to the crystalline structure of ZnO, remained with the formation of ZnO@ZIF-8, which can be represented by the peaks at positions 36.66°, 56.36°, 63.12°, and 68.44° 2θ, referring to the planes of symmetry (102), (110), (103), and (112), respectively, of the wurtzite hexagonal structure (JPCDS card number: 36-1451) [23].

Figure 2B shows the FTIR spectra of the ZIF-8 and ZnO@ZIF-8 NPs. It can be observed that all samples present very high similarities. According to Feng et al., at higher frequencies, ZIF-8 showed two small stretch bands present at 3132 and 2926 cm^−1^, which can be attributed to the C-C vibrational stretching of the imidazole ring and the methyl group present in the ligand, respectively. The band at 1585 cm^−1^ can be attributed to C=N, while the bands at 1430 and 1313 cm^−1^ correspond to the elongation of the entire ring. Some bands, between 1350 and 900 cm^−1^, can be attributed to the angular flexion of the ring, and bands 763 and 692 cm^−1^ can be related to the aromatic C-H elongation. The band present at 421 cm^−1^ corresponds to the Zn-N stretch with zinc in the ZIF-8 structure, which binds to 2-MeIM nitrogen atoms during the formation of ZIF-8. On the other hand, the band at 482 cm^−1^ corresponds to the formation of the Zn-O bond [24], indicating the presence of ZnO in the material. However, due to the encapsulation of the ZnO within the structure of ZIF-8, the rest of the characteristic bands of ZnO were probably masked, which suggests that ZIF-8 is providing additional protection for the nanoparticle.

**Figure 3:**
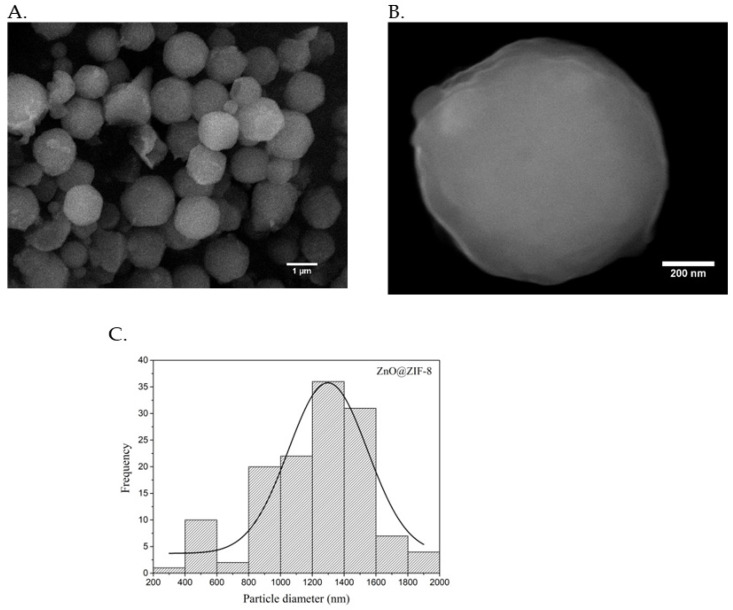
SEM images (**A**,**B**) and particle size distribution (**C**) of ZnO@ZIF-8 particles.

The morphological structure and size distribution of the ZnO@ZIF-8 particles were analyzed by scanning electron microscopy (SEM), as shown Figure 3.

The SEM images show that the formed particles are homogeneous and spherical. It is possible to evidence an average particle size of 1.29 ± 0.45 μm, which is larger than the pure ZnO previously reported, including that synthesized by our group using the sol–gel method (average size of 5 nm) [21,25], suggesting the association of these two materials as one in the same particle.

### 3.2. Loading of CIP

To evaluate the potential application of ZnO@ZIF-8 as the antimicrobial system synthesized and characterized herein, the conventional antimicrobial drug ciprofloxacin was loaded onto ZnO@ZIF-8 by an impregnation method [26]. 

After 12 h, there was an average concentration of drug incorporation of 46%, which is a good percentage of incorporation when related to other studies in the literature, mainly of poorly soluble drugs [26,27]. There was no statistically significant difference between the incorporation times of 12, 24, and 48 h, using the ANOVA test. Therefore, for future CIP incorporation assays in the ZnO@ZIF-8 MOF system, 12 h of incorporation in ethanol are sufficient to reach the plateau of loading. 

The previous data reported in the literature [28] show that ZIF-8 exhibited low values of drug adsorption with other antimicrobials, e.g., 6.73% of vancomycin. Therefore, these results confirm the excellent capacity of ZIF-8, even with ZnO, to incorporate a large amount of CIP.

**Figure 4:**
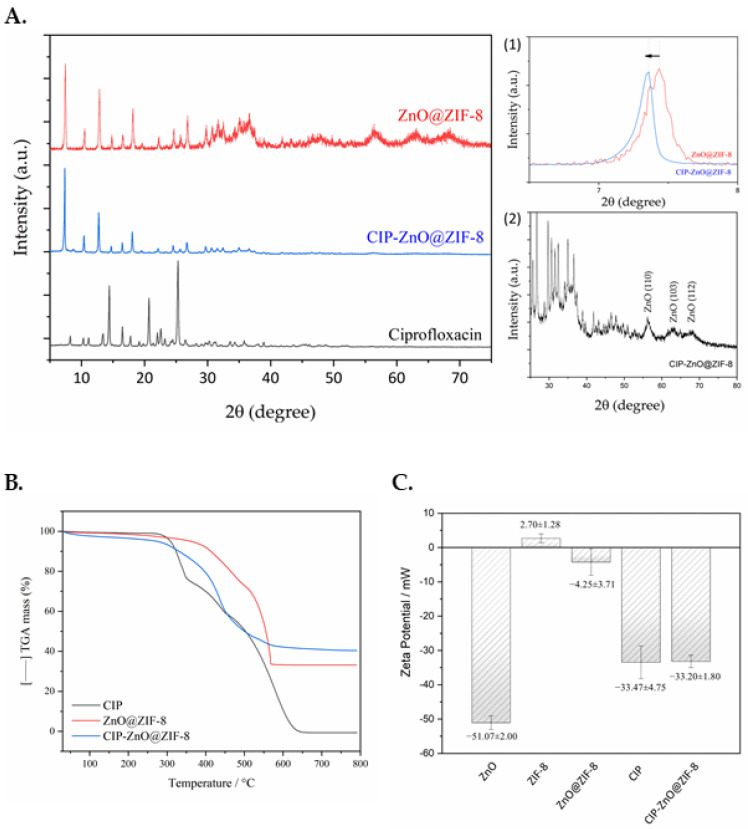
XRD analysis of CIP-ZnO@ZIF-8 after incorporation assays (**A**), with shift peak identification (**A1**) and identification of ZnO after incorporation (**A2**); TGA analyses of CIP, ZnO@ZIF-8, and CIP-ZnO@ZIF-8 (**B**); Zeta potential measurements for ZnO, ZIF-8, ZnO@ZIF-8, CIP, and CIP-ZnO@ZIF-8 NPs (n = 3) (**C**).

After the loading experiments, all characterizations of the XRD, TGA, and Zeta potential results confirmed that CIP was present, and, besides that, the structural integrity of MOF and ZnO was maintained after the drug encapsulation. XRD analysis (Figure 4A) showed that, after drug incorporation, the crystal structure of ZIF-8 remained unchanged. However, some peaks shifted slightly to the left (Figure 4A1), suggesting that the pore crystal structure of ZIF-8 may have changed during the incorporation of ciprofloxacin [29,30,31], which may evidence the entry of the drug into the pores of ZnO@ZIF-8. The crystallinity index was studied and calculated by the Segal equation [32]. The ZnO@ZIF-8 sample has a lower crystallinity than the sample with the encapsulated drug, approximately 30.23% for pure ZnO@ZIF-8 MOF compared to 61.57% after encapsulation (CIP-ZnO@ZIF-8). Such a factor happens in disordered nanocrystalline materials that depend on particle size, though they are not totally amorphous [33,34]. The differences between the diffractograms obtained from the samples before and after encapsulation are mainly due to the presence of the drug in the pores of the structure, which strongly affects the crystallinity of ZIF-8, making the MOF more structurally stable [35,36,37]. Another factor to be considered is that the absence of diffraction peaks, characteristic of the CIP drug, evidences the predominant entry of the drug into MOF [38].

The TGA data (Figure 4B) provide information on the possible proportion of each component after the incorporation of CIP into MOF ZnO@ZIF-8. The TG data from the ZnO@ZIF-8 sample have two steps of mass loss. The first step occurs in the range from 30 to 371 °C (∆mTG = 5.19%). As the loss is relatively low, up to 105 °C, it can be presumed that the ZnO@ZIF-8 sample was effectively activated, so this step can be related to the loss of the GPTMS molecules that covered the ZnO. The second step of mass loss (∆mTG = 63.73%) is associated with the thermal decomposition of the organic fraction of ZIF-8 in the range of 303.16–576.86 °C, which occurs in a single step followed by the formation of ZnO as a residue, corresponding to 33.29% of the total mass of the sample. Hence, ZIF-8 is stable up to 300 °C. The TG data of the CIP-ZnO@ZIF-8 sample show the presence of three steps of mass loss. The first step occurs between 30 and 261 °C (∆mTG = 4.75%), with accentuated loss at 105 °C, which may refer to the release of the solvent adsorbed on the nanostructure, mainly by water and residual ethanol. The second step, which occurs from 260 °C to 481.89 °C (∆mTG = 42.5%), corresponds to the possible degradation of the drug incorporated into the MOF, since the event’s starting temperature corresponds to the degradation temperature of the pure CIP, in the range of 287–372 °C. The third step of mass loss takes place in the region of 481.89–626.02 °C (∆mTG = 11.55%) and can be associated with the decomposition of the organic fraction of ZIF-8 (2-MeIM), followed by the formation of ZnO as the main product of the thermal decomposition of the sample (40.25% in mass).

The surface charge of the produced nanoparticles was also studied by the Zeta potential. The results are shown in Figure 4C. It can be observed that, in the results, all NPs, except ZIF-8, presented a negative surface charge. The surface charge of ZnO, which originally had its surface modified with GPTMS, has a value of −51.07 mV; this is consistent with its colloidal stability in water, which favors the production of the outer layer, entirely of ZIF-8, in addition to a good size control, as presented in the SEM results. When the outer layer of ZIF-8 is formed, the surface charge of NP approaches 0 (ZP = −4.25 mV), which is in accordance with the result shown by the synthesis of ZIF-8 (ZP = 2.70 mV), in addition to confirming the literature data for the formation of ZIF-8 [39]. The formation of ZIF-8 on the surface of ZnO decreases colloidal stability in water but does not change the aggregation state of NPs, as shown in the SEM. From the incorporation of CIP, the surface charge of the NPs of CIP-ZnO@ZIF-8 (ZP = −33.20 mV) presents a Zeta potential similar to the surface charge of CIP alone (ZP = −33.47 mV). These data suggest that CIP has been successfully incorporated into MOF, that the drug is on the surface, and that there is the formation of nanoparticles with excellent colloidal stability. 

**Figure 5:**
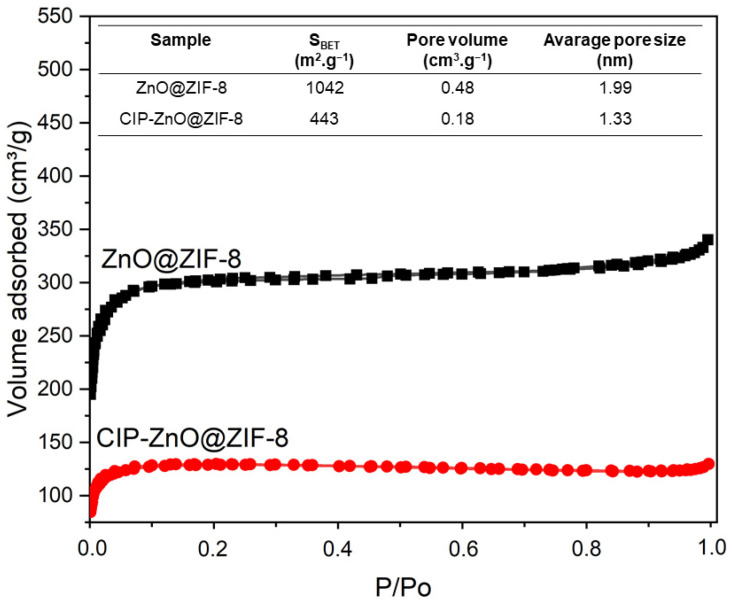
N_2_ adsorption–desorption isotherms and BET surface areas (inset table) of ZnO@ZIF-8 and CIP-ZnO@ZIF-8.

The porosity of the ZnO@ZIF-8 NPs was also analyzed by N_2_ porosimetry, comparing before and after encapsulation, as shown in Figure 5.

The porosity of the ZnO@ZIF-8 showed a decrease in the specific surface area of the MOF, which can be justified by the incorporation of a large amount of CIP. In addition, the incorporation of the drug also caused a stronger decrease in pore volume, as expected.

### 3.3. In Vitro CIP Release

**Figure 6:**
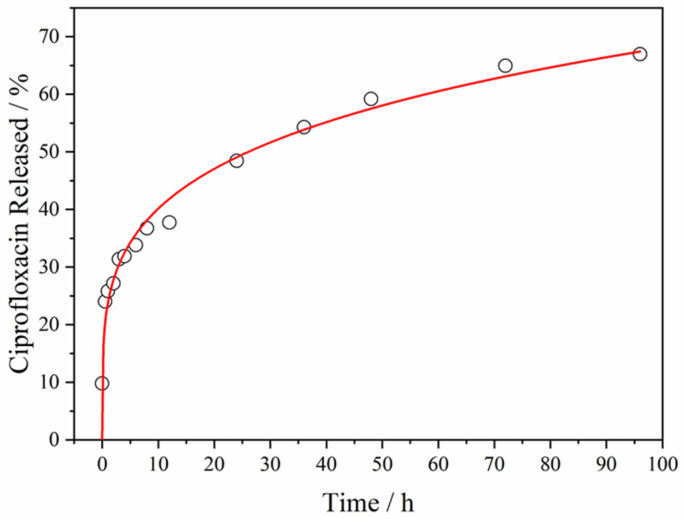
Profile of CIP release from ZnO@ZIF-8 into PBS solution at pH 7.4, at 37 °C. The data were fit to the Korsmeyer–Peppas equation, as indicated by the red line.

The UV spectra were obtained for each material released at different time points, performing the corresponding calculations and creating the in vitro release profile of CIP from ZnO@ZIF-8 in PBS medium at pH 7.4, as shown in Figure 6.

In detail, a tiny burst release at the early stage of the delivery can be observed, with around 10% of the CIP released from the ZnO@ZIF-8 within the first hour. The trial has a high rate of release in the first 12 h, with 37% of the drug released. Although the last times collected after 72 and 96 h were not statistically different (*p* > 0.05), the behavior of the graph shows that desorption still increased after the end of the assay. After 4 days of experimentation, 67% of the drug was released. The prolonged release of CIP may be interesting for application as an antimicrobial treatment, suggesting that the treatment can be performed without the need for repeated doses.

The kinetic CIP release profile was fit by mathematical models, using the Sigma Plot 10.0 software (Systat Software, San Jose, CA, USA), to understand the mechanism underlying the drug release. Table 1 summarizes the drug release models and the respective fitting equations and parameters of regression for each equation. Zero-order kinetics models were performed for diffusion-control release kinetics of the drug [40]. The Korsmeyer–Peppas kinetics model was described for the release of mechanisms with diffusion, matrix swelling, and surface erosion [41]. The Hixson–Crowell model also studies the erosion surface, describing the decomposition of the material into smaller pieces [42]. The first-order kinetic model describes hydrolytic-degradation behaviors [43].

The CIP release from ZnO@ZIF-8 showed a strong correlation with the Kosmeyer–Peppas model. The value of ƞ ≤ 0.45 represents cases of Fickian transport, indicating that diffusion controlled the release process [44]. The diffusion capacity is a constant of proportionality, which can be established as a function of the concentration or physical variables that are related to the structure of the material, such as the existence of pores between the chains of ZIF-8, which allows drug diffusion. Therefore, the drug release from ZnO@ZIF-8 is directly related to CIP desorption from the pores and drug diffusion through the frameworks. 

### 3.4. Antimicrobial Activity

MIC determination was performed by the XTT assay, and the results are described for the determination of the antimicrobial action of ZIF-8, ZnO, ZnO@ZIF-8, and CIP-ZnO@ZIF-8 against strains of *S. aureus* (Table 2) and *P. aeruginosa* (Table 3) bacteria.

According to the results presented, the concentration of the bacteria used in the tests was maintained at 10^4^ CFU mL^−1^, and the concentration of the samples used at the beginning of the serial dilution of base 2 was 1 mg mL^−1^ and 5 mg mL^−1^ for the *S. aureus* strain and the *P. aeruginosa* strain, respectively. Results are shown after 24 h of incubation protected from light. ZIF-8 alone did not demonstrate any antimicrobial activity against both analyzed bacteria, and this is expected for the ZIF-8 antimicrobial effect [45].

However, the analysis of the results against the *S. aureus* strain in Table 2, ZIF-8 in association with ZnO, shows a MIC of 0.062 mg mL^−1^, demonstrating a similarity with the results obtained with pure ZnO; that is, even with the formation of ZIF-8, ZnO was able to produce the antimicrobial action against the *S. aureus* strains. Furthermore, the MOF ZnO@ZIF-8, associated with the antibiotic ciprofloxacin, resulted in a MIC 10 times lower than that of MOF alone, of 0.0062 mg mL^−1^, after 24 h of incubation. Some of the literature showed a MIC of pure CIP against *S. aureus* that was greater than that presented in this study. Kwak et al. presented a MIC that was higher than 0.128 mg mL^−1^ for CIP alone [46]. Another work, carried out by Bazzar et al., studied a MIC of CIP ranging from 0.005 to 0.08 mg mL^−1^ [47]. Masadeh et al. [48] showed a MIC for *S. aureus* and *P. aeruginosa* of 3.42 µg mL^−1^ and 5.4 µg mL^−1^, respectively, but the concentration of the ciprofloxacin that was used (100 µg mL^−1^) was much higher than the concentration released in our work. Thus, it is possible to conclude that the association of CIP with ZnO@ZIF-8 promoted a decrease in the amount of CIP needed to obtain a MIC similar to that of the pure ciprofloxacin drugs.

On the other hand, when analyzing the results in Table 3, regarding the antimicrobial action against strains of *P. aeruginosa*, a MIC of 2.5 mg mL^−1^ was observed for ZnO@ZIF-8, which is five times greater than when the ZnO nanoparticles are pure (0.5 mg mL^−1^); this can be explained by the lower action of ZnO on Gram-negative bacteria. In addition, ZIF-8 provided resistance to ZnO release to the medium and, consequently, reduced the antimicrobial action. Furthermore, ZIF-8 has low action against Gram-negative bacteria [45,49]. 

The incorporation of CIP showed a substantial gain in action against Gram-negative bacteria. The presented MIC of 0.0125 mg mL^−1^, against *P. aeruginosa*, showed a 200-fold increase in the antimicrobial activity of the complex, in addition to a similar result against Gram-positive bacteria. In the literature, with an increase in ciprofloxacin concentration in the presence of ZnO, there is an improvement in antimicrobial activity against *P. aeruginosa* [13].

The antimicrobial activity of ZnO@ZIF-8 was reported elsewhere, by Redfern et al. [50]. However, in that work, the structure of ZnO@ZIF-8 was formed by a core of ZIF-8 with the formation of ZnO nanorods on its surface; therefore, this occurred without the premise of forming a drug nanocarrier. In our study, it was demonstrated that the ZnO@ZIF-8 structures have a similar MIC, though with the possibility to modify the release of a drug and optimize the ciprofloxacin antimicrobial activity against *S. aureus*.

ZnO has important antimicrobial activity, which is due to a series of mechanisms of toxicity for bacteria, mainly through the generation of reactive oxygen species (ROS) and the release of Zn^2+^ ions [51]. The release of metal ions can inhibit some enzymes and affect the bacterial respiratory chain [52], and the release of Zn^2+^ is dependent on the size and morphology of the synthesized ZnO. The smaller the size of the formed ZnO nanoparticles is, the greater the surface area of interaction with the solvent is, and the greater the ease of the Zn^2+^ release and the ease of the transport of these components into the bacterium are [53]. Furthermore, the ZIF-8 structure may also play an important role in the release of Zn^2+^ ions for antimicrobial activity [54]. As the ZnO core formed is the same compared to the formation of ZnO nanoparticles and the ZnO core in the formation of ZnO@ZIF-8 NPs, the similarity of structures and size may explain the similarity in antimicrobial activity against *S. aureus* in the presented results.

On the other hand, the formation of ROS by ZnO, from exposure to ultraviolet light, leads to the formation and accumulation of singlet oxygen, hydroxyl radical, hydrogen peroxide, and superoxide anions, among others. Reactive oxygen species can damage the internal components of bacteria, such as proteins and DNA [52]. Among these components, hydrogen peroxide easily penetrates the bacterial membrane due to its physicochemical properties, resulting in the destruction of cellular components, such as lipids, proteins, and DNA, leading to the death of the bacteria [53].

## 4. Conclusions

In the present work, it was possible to successfully produce the compound ZnO@ZIF-8 with a new adjusted method that managed to maintain the physicochemical properties of ZnO and the formation of MOF ZIF-8, by successfully incorporating the antimicrobial ciprofloxacin, with the aim of exploiting this MOF conjugate’s high loading capacity, a controlled drug release, and an antibacterial activity that can act on Gram-positive and Gram-negative bacterial strains. The new synthesis, not previously reported in the literature, brings the advantages of controlling ZnO@ZIF-8 particle size and providing good nanocarrier properties. The antibacterial activity shows that CIP-ZnO@ZIF-8 has good activity against strains of *S. aureus* and *P. aeruginosa*, with a lower dosage of ciprofloxacin than other studies in the literature.

One of the greatest challenges of science is to escape the mechanisms of microbial resistance [55]. Antimicrobials act in a specific way, by inhibiting the components that maintain microbial metabolism. However, when using nanomaterials such as ZnO, as discussed above, antimicrobials target pathways where antibiotics may fail. This is possible because the most antibiotic-resistant mechanism does not have the same pathways in which nanoparticles act [56]. Furthermore, ZnO NPs possess several simultaneous antimicrobial mechanisms. In this sense, the developed material CIP-ZnO@ZIF-8 can be an alternative to minimizing microbial resistance, by combining several mechanisms of activity and being an innovative system that can still be used to incorporate several other molecules.

## Figures and Tables

**Table 1:** Kinetic models used for analysis of CIP release from ZnO@ZIF-8 and their respective calculated parameters.

Model	Equation ^a^	*k*	ƞ	R²
Zero Order	*F* = *kt*	0.972 ± 0.177	-	−1.06
Korsmeyer–Peppas	*F* = *kt^n^*	23.682 ± 1.352	0.223 ± 0.016	0.96
Hixson–Crowell	*F* = 100[1 − (1 − *kt*)^3^]	0.006 ± 0.001	-	−0.48
First Order	*F* = 100(1 − *e*^−*kt*^)	0.615 ± 4.324	-	−0.26

^a^*F* represents the cumulative drug release percentage; *t* represents the time of release; *k* is the release rate constant; and ƞ is the release exponent that corresponds to the transport mechanism.

**Table 2:** MIC of samples against *S. aureus* (Gram-positive bacteria) (n = 3).

Sample	MIC
ZIF-8	>1 mg mL^−1^
ZnO	0.0652 mg mL^−1^
ZnO@ZIF-8	0.0625 mg mL^−1^
CIP-ZnO@ZIF-8	0.0062 mg mL^−1^

**Table 3:** MIC of samples against *P. aeruginosa* (Gram-negative bacteria) (n = 3).

Sample	MIC
ZIF-8	>5 mg mL^−1^
ZnO	0.5 mg mL^−1^
ZnO@ZIF-8	2.5 mg mL^−1^
CIP-ZnO@ZIF-8	0.0125 mg mL^−1^

## Data Availability

Not applicable.
